# Supplementary material for: An ecosystem for producing and sharing metadata within the web of FAIR Data
Source: Gigascience. 2025 Jan 8;14:giae111. doi: 10.1093/gigascience/giae111 (PMC11707607; doi:10.1093/gigascience/giae111)
Supplement: giae111_GIGA-D-24-00167_Revision_1 [file giae111_giga-d-24-00167_revision_1.pdf]

GigaScience

# Maggot: An ecosystem for sharing metadata within the web of FAIR Data

--Manuscript Draft--

| | |
|---|---|
| **Manuscript Number:** | GIGA-D-24-00167R1 |
| **Full Title:** | Maggot: An ecosystem for sharing metadata within the web of FAIR Data |
| **Article Type:** | Technical Note |
| **Funding Information:** | Agence Nationale de la Recherche (ANR-11-INBS-0010) — Mr Daniel Jacob<br>Agence Nationale de la Recherche (ANR-21-CE21-0014) — Mr Daniel Jacob |
| **Abstract:** | Background<br><br>Descriptive metadata is vital for reporting, discovering, leveraging, and mobilising research datasets. However, resolving metadata issues as part of a data management plan can be complex for data producers. To organise and document data, various descriptive metadata must be created. Furthermore, when sharing data, it is important to ensure metadata interoperability in line with FAIR principles. Given the practical nature of these challenges, there is a need for management tools that can assist data managers effectively. Additionally, these tools should meet the needs of data producers and be user-friendly, requiring minimal training.<br><br>Results<br><br>We developed Maggot (Metadata Aggregation on Data Storage), a web-based tool to locally manage a data catalogue using high-level metadata. The main goal was to facilitate easy data dissemination and deposition in data repositories. With Maggot, users can easily generate and attach high-level metadata to datasets, allowing for seamless sharing in a collaborative environment. This approach aligns with many data management plans as it effectively addresses challenges related to data organisation, documentation, storage, and the sharing of metadata based on FAIR principles within and beyond the collaborative group. Furthermore, Maggot enables metadata crosswalks, i.e., generated metadata can be converted to the schema used by a specific data repository or be exported using a format suitable for data collection by third-party applications.<br><br>Conclusion<br><br>The primary purpose of Maggot is to streamline the collection of high-level metadata using carefully chosen schemas and standards. Additionally, it simplifies data accessibility via metadata, typically a requirement for publicly funded projects. As a result, Maggot can be utilised to promote effective local management with the goal of facilitating data sharing while adhering to the FAIR principles. Furthermore, it can contribute to the preparation of the future EOSC FAIR Web of Data within the European Open Science Cloud framework. |
| **Corresponding Author:** | Daniel Jacob<br>INRAE Nouvelle-Aquitaine Bordeaux Centre: Institut National de Recherche pour l'Agriculture l'Alimentation et l'Environnement Nouvelle-Aquitaine Bordeaux Centre<br>Villenave d'Ornon, FRANCE |
| **Corresponding Author Secondary Information:** | |
| **Corresponding Author's Institution:** | INRAE Nouvelle-Aquitaine Bordeaux Centre: Institut National de Recherche pour l'Agriculture l'Alimentation et l'Environnement Nouvelle-Aquitaine Bordeaux Centre |
| **Corresponding Author's Secondary Institution:** | |
| **First Author:** | Daniel Jacob |
| **First Author Secondary Information:** | |

*Powered by Editorial Manager® and ProduXion Manager® from Aries Systems Corporation*

| | |
|---|---|
| **Order of Authors:** | Daniel Jacob |
| | François Ehrenmann |
| | Romain David |
| | Joseph Tran |
| | Cathleen Mirande-Ney |
| | Philippe Chaumeil |
| **Order of Authors Secondary Information:** | |
| **Response to Reviewers:** | Dear Editor,<br><br>Concerning manuscript " Maggot: An ecosystem for sharing metadata within the web of FAIR Data " (GIGA-D-24-00167), all remarks of reviewers have been considered.<br>The reviewers noted a lack of detail and clarity, with the manuscript appearing too general. This issue stemmed from our initial intent to write a longer and more comprehensive article. However, we ultimately decided to focus on a more technical piece. The version originally submitted unfortunately reflected this change in direction inadequately. In the revised version, we have fully embraced the technical focus, ensuring that any overly general arguments have been removed unless they explicitly support our approach.<br>Reviewers, experts in the field, provided us with valuable revision elements to improve the manuscript. We were keen to satisfy the various advice and remarks, especially since they were provided in a spirit of benevolence with a real interest for the content of the manuscript.<br>The answers and changes that have been made are detailed below.<br>Additionally, we submitted the revised manuscript to a native English speaker for a thorough language review.<br>Please let us know if some additional modifications are needed.<br>Best wishes,<br><br>Daniel Jacob<br><br><br><br>Reviewer reports:<br><br>Reviewer #1:<br><br>* The authors describe in very general terms a system to assist in the creation and management of experimental metadata.  The paper provides a thoughtful description of how a metadata-management system needs to fit into an overall data ecosystem, tracing the flow of requirements from data-management plans to the archival of results in an online repository.  The authors do not provide enough information about their system, however, for the reader to understand what has been accomplished.  What does the system look like?  Who has used it?  What has the system done well?  What have the problems been?  What future work is planned to address the problems?  In the absence of more information about the system—its architecture, its user interface, and its metadata model—and in the absence of any evaluation of the system, it's hard to know what the reader should take away.<br><br>Answer: We completely agree with your observations. As explained to the editor, our initial plan was to write a longer and more general article, but we ultimately decided to focus on a more technical piece. Unfortunately, the originally submitted version reflects this shift in direction poorly. In the revised and corrected version, we have fully embraced the choice of a technical article. However, in the initial version, we presented certain aspects inadequately, and the online documentation could have provided additional information.<br>In this revised version, we now fully explain our choices in the “Design Considerations” section, particularly regarding the metadata schema (lines 150-161). In the “Results” section, we now describe in detail the different stages of the configuration and provide recommendations concerning the choice of metadata, as well as an approach to the selection of controlled vocabularies. Furthermore, the three main functionalities are |

now described with two figures (Figures 3 and 4) used to illustrate them.

* There are open questions regarding general-purpose metadata models (such as the Dataverse model, the DataCite schema, DCAT, and DDI), to which the authors appear to be committed, and the relationship of these general frameworks to discipline-specific metadata standards (such as those available through Fairsharing.org). The FAIR Guiding Principles come down on the side of discipline-specific metadata standards, and the authors would seem to be well positioned to contribute to dialog in this regard.

Answer: This is a very interesting topic indeed, and it was the subject of the initial article. However, in this technical article, we focus on our choice based more on practical reasons (national repository based on Dataverse / DDI) than a discussion on the good criteria for choosing a metadata schema. That said, since the Maggot software allows users to select their own schema, such a discussion could be the subject of a separate article. As noted in our revised conclusion, we want to create a community around this tool, and these questions will be for sure at the heart of our concerns.

* The paper surprisingly has no discussion of related work. An advantage of open review is that I, as a referee, do not need to feel sheepish in suggesting that my own work is probably the most relevant here. My laboratory has been working on a system called CEDAR for nearly ten years. CEDAR has been adopted by several large scientific consortia, and it is now integrated into the data/metadata-uploading facilities of generalist data repositories such as Dryad and OSF. I'd be happy to discuss CEDAR with the authors outside of this review. One can read more about our work here: https://www.nature.com/articles/s41597-022-01815-3

Answer: We did not consider it necessary to include a discussion section. However, in this revised version, we justify our choices in relation to other approaches (all types of metadata in a single data warehouse - e.g. CEDAR, FAIRDOM-SEEK, MetaboLights vs. metadata distributed across multiple repositories) in the "design considerations" section (lines 150-161).
Thank you for the reference which we read with interest and have suggested to our potential readers
Indeed, the CEDAR system seems to us to be very relevant for the creation of metadata and data management in a spirit of open scientific data, following the FAIR principles. Although we have not been able to evaluate it in practice, our experience with numerous data producers suggests that the more exhaustive a system aims to be, the more reluctance there is to document the data. Unfortunately, many people prefer to deposit OMICS data directly into a general repository, even though there are more relevant specialised repositories available. This demonstrates that many of us are still far from fully embracing open data management in line with the FAIR principles. We emphasise at the start of the “Background” section the need to “reward the good actors” if we want this to progress.

* The authors may not be aware, but the word "maggot" has a rather unfortunate meaning in English.

Answer: It is a point of view. It becomes difficult to find a name that is a little original. So, this one stands out. Shouldn't the maggots be rehabilitated? ;-) Besides, in French, "un mago" is slang for a lucky find or a great discovery—so maybe we are just ahead of the curve on this one!

Reviewer #2:

* The reviewer thank the authors for their work, which presents a set of software components working together for the creation, editing and sharing of metadata. All code is made available via github code repository and is supported by extensive documentation.

Answer: We are very pleased that you highlight all our work. Thanks.

Main comments:

* A large amount of work and engineering went into the Maggot ecosystem. The communication could however be improved by clarifying the narrative.
The focus of the Maggot is 'generic metadata' reporting as opposed to domain specific metadata (e.g., metadata specific to report experimental details, instrumentation specifics, experimental results).

Answer: Indeed, it is one of the main focuses. In this revised article, we preferred the adjective “high-level” instead of generic, general, or even descriptive. This helps to better emphasise the fact that this metadata is like one of the “hats” on a dataset.

* In the 'Design Consideration' section, the authors highlight their structured approach starting with descriptor identification, terminology definition or adoption and finally standard selection.
Can the authors clarify the following sentence "Rather than mandating data conformity, the schema should be flexible to accommodate pre-existing data” in the second paragraph of that section?

Answer: This sentence means that it is more important to consider reality, in this case existing data, than standards with which we must willy-nilly deal. This sentence was a bit clumsy. It no longer appears in the revised version.

* In the following section about 'controlled terminologies', can Maggot be set to terminology services based on Bioportal? for instance, is it possible to interact with EMBL-EBI Ontology Lookup Service or to a custom service? What would be the technical requirements? It might be helpful to provide a pointer to Maggot documentation on that topic.

Answer: Ontologies based on OntoPortal (not only BioPortal, but also AgroPortal for example) are natively supported in Maggot. Additionally, we have implemented a mechanism based on Twitter's Typeahead library, allowing Maggot to support almost any controlled vocabulary accessible via API. For instance, the APIs for the SKOSMOS thesauri are supported in this way; they were managed specifically before but less well. Moreover, the EBI ontologies via OLS are also supported via this library. This approach allows for harmonising the use of various API-based vocabulary services, including OntoPortal APIs. An addition has been made to the online documentation regarding this and template scripts are provided with the source code for ease of implementation. See https://inrae.github.io/pgd-mmdt/definitions/vocabulary/
A test version is available online: (https://pmb-bordeaux.fr/maggot-vt/entry). In the “Descriptors” section, the CVs concerning the field “Topic Classification” are based on the thesaurus-inrae (SKOSMOS) and those concerning the fields “Experimental factor”, “Type of measurement” and “Type of technology” are based on OLS.

* In the next paragraph, as the authors argue for raising awareness in existing resources, pointing to resources such as FAIRSharing.org, Re3data or Bioregistry.org would be useful.

Answer: This paragraph has been shortened in the revised version, and placed at the end of the section. We have also added these resources (lines 188-191). Although, they are more useful to data managers and data stewards than to data producers.

* In the following section, can the authors specify provide an example of Core Trust seal repository have targeted ? Similarly, in the case of submitting metadata via API, which schema have need chosen or can be selected (e.g., datacite, dcat )?

Answer: We have removed this sentence related to the Core Trust seal repository in the revised version. No longer applicable.
Furthermore, in the case of submitting metadata via API, it is the metadata crosswalks definition file that defines the chosen schema compliant with the targeted repository. This is now better explained in the subsection "Metadata crosswalks definition" (lines 244-261).

* What is " a metadata crossing approach based on the mapping defined upstream by the data manager"? can it be simplified by saying that 'mappings are available between

Powered by Editorial Manager® and ProduXion Manager® from Aries Systems Corporation

selected models"? How many models have been mapped and are available out of the box?

Answer: This sentence is not clear, indeed. So, we rewrote it: “These functionalities use a metadata crosswalk approach based on the mapping files defined at this second level. These mapping files map the metadata defined at the first level to the output metadata schema (Fig. 1)” (lines 252-255).

* Providing a pointer to the extensive maggot documentation would be a nice addition: https://inrae.github.io/pgd-mmdt/chats/chat4/

Answer: Ok, good suggestion. Done (line 253)

* The last paragraph of the section seems to somehow contradict an earlier section claiming that "creating metadata should not compel data producers to engage in training in topics or concepts outside their expertise, such as FAIR principles, the semantic web, or metadata schema, unless they choose to do so" with the following sentence "Unavoidably, the development and dissemination of metadata, involving a metadata-centric culture, underline the need for ongoing training initiatives and data stewards are pivotal in educating data producers".

Answer: By "metadata-centric culture" we mean that users must be fully aware that without metadata, their data has little or no use. It is an inseparable whole. So every time we mention data it should also imply the metadata without having to specify it. On the other hand, users do not necessarily need to know concepts such as the notion of metadata schema, or even the FAIR principles. Not to mention the semantic web and other Link Data. In this revised version, we did not wish to develop this point, so only a small paragraph remains at the end of the "Design considerations" section (lines 184-191).

*Result section:

* s/supporting the wide data diversity range within a collective./ supporting a community's varied annotation needs/

Answer: Ok corrected (line 118).

* The first 2 sentences of the third paragraph to section may be at ease in the design consideration section, as it describes to decoupling a storage and metadata creation.

Answer: Ok we agree with this suggestion. Done (lines 134-148).

* The authors introduced Maggot as tool specifically targeting 'generic metadata'. However, in the following sentence, they state " Maggot supports the input of both descriptive and administrative metadata for any type of data, including datasets, images, sequences, and more, with customizable field definitions to suit diverse user requirements. Moreover, Maggot emphasises ease of use and adaptability." How can users know when they cross over into domain specific metadata?

Answer: In the revised version, we have removed this ambiguity by focusing only on high-level metadata - see the second paragraph of the "design considerations" section (lines 126-132). Also, there should be no question of metadata specific to a particular data type. It is nevertheless still possible to define such metadata, but we believe that this would be a misuse of Maggot.

* ODAM appears in figure 2. Can the authors provide more information about the relation with Maggot and ODAM when it comes to metadata description. Can Maggot be used to index metadata declared in ODAM or is maggot solely concerned with generic metadata about the ODAM resources (authors, owner, organization, terms of use)?

Answer: Good suggestion. We have added a paragraph at the end of this section explaining the connection between ODAM and Maggot (lines 328-336).

Powered by Editorial Manager® and ProduXion Manager® from Aries Systems Corporation

* In the fourth paragraph on the section, it could be beneficial to introduce the selection of OAI-PMH earlier and then explain the benefit it provides in terms of long term preservation and/or migration away from Maggot.

Answer: Ok. We added some words to this effect in the "Metadata crosswalks definition" paragraph (lines 248-257).

* Does Maggot support the export of metadata as schema.org based json-ld payloads natively?

Answer: Yes, it does. But on condition of having defined the mapping file ensuring the metadata crosswalk to the desired schema. In the mapping proposed by default, we base ourselves on schema.org. But it would be entirely possible to choose another scheme like DublinCore.

*Implementation and Documentation section:

* Can the authors expand on the issue of access to controlled vocabularies via APIs? In particular, if any caching mechanism is in place and if different versions of specific vocabularies can be fetched or specified?

Answer: Ok. We have now clarified these points in this section (lines -359-365).

* The authors should be commanded for the extensive mkdocs based documentations. It may be nice to amend some of the figures (e.g., figure 3, https://inrae.github.io/pgd-mmdt/publish/) so they can highlight the availability of that excellent resource for the readership

Answer: Ok we agree with this suggestion. Done. The Fig.3 becomes now the Fig. 1 in the revised version.

*Conclusion Section:

* The first sentence feels a bit too long. break in 2 and push the «frictionless FAIR metadata sharing' to another sentence. the authors should be mindful of using 'frictionless' in this context due to use of frictionless data framework in ODAM, as a possible source of confusion. suggestion = s/frictionless/native/

Answer: OK the first sentence has been rewritten (lines 374-376).

* The last sentence of the first section leads to the following question: Does maggot allow granular specification of terms of uses (i.e, beyond the provision of a uri to a license document) ? If so, it would nice to add a figure or code snippet showing how this is done.

Answer: The point was indeed intended to be more general. To maintain focus on the scope of the tool, this sentence has been deleted. Maggot specifically allows you to specify conditions of use for data associated with high-level metadata. However, it doesn't prevent specific conditions of use from being specified for each type of data deposited in other data repositories.

Minor comments:

* Title: What is the "web of FAIR data"? could be the title be simplified to "Maggot : ecosystem for producing and sharing metadata to support FAIR data practice "?

Answer: The suggestion to add production in the title suits us completely. As for the "Web of FAIR data", we base ourselves on the objectives set by the EOSC: see https://beopen-project.eu/storage/files/parti-2-eosc-a-web-of-fair-data-k-luyben.pdf

* Abstract.background: order -> 1/reporting, 2/discovery, 3/mobilisation. then "poses challenges to data producers"

| | |
|---|---|
| | Answer: OK we agree with this suggestion. Done.<br><br>* s/" in alignment with FAIR principles/"in accordance to the FAIR principles"/<br><br>Answer: OK corrected<br><br>* Abstract.results: "Comprehensible" - some ambiguity here as to where it should be "comprehensive" or "explicit"?  Then a question about "Collaborative environment" . Does it mean that Maggot allows concurrent editing of the same document by multiple users (as in a Google document for instance)?<br><br>Answer: Not really. We rewrite the sentence as : "With Maggot, users can easily generate and attach high-level metadata to datasets, allowing for seamless sharing in a collaborative environment." (lines 37-38).<br><br>* s/for enabling/ to enable/<br><br>Answer: OK corrected<br><br>Thank you for all the criticisms and suggestions which helped clarify the narrative. Very appreciated! |
| **Additional Information:** | |
| **Question** | **Response** |
| Are you submitting this manuscript to a special series or article collection? | No |
| **Experimental design and statistics**<br><br>Full details of the experimental design and statistical methods used should be given in the Methods section, as detailed in our Minimum Standards Reporting Checklist. Information essential to interpreting the data presented should be made available in the figure legends.<br><br>Have you included all the information requested in your manuscript? | No |
| If not, please give reasons for any omissions below.<br><br>as follow-up to "**Experimental design and statistics**<br><br>Full details of the experimental design and statistical methods used should be given in the Methods section, as detailed in our Minimum Standards Reporting Checklist. Information essential to interpreting the | Not applicable |

| | |
|---|---|
| data presented should be made available in the figure legends.<br><br>Have you included all the information requested in your manuscript?<br><br>" | |
| **Resources**<br><br>A description of all resources used, including antibodies, cell lines, animals and software tools, with enough information to allow them to be uniquely identified, should be included in the Methods section. Authors are strongly encouraged to cite Research Resource Identifiers (RRIDs) for antibodies, model organisms and tools, where possible.<br><br>Have you included the information requested as detailed in our Minimum Standards Reporting Checklist? | Yes |
| **Availability of data and materials**<br><br>All datasets and code on which the conclusions of the paper rely must be either included in your submission or deposited in publicly available repositories (where available and ethically appropriate), referencing such data using a unique identifier in the references and in the "Availability of Data and Materials" section of your manuscript.<br><br>Have you have met the above requirement as detailed in our Minimum Standards Reporting Checklist? | Yes |

Manuscript

Click here to access/download;Manuscript;Maggot_v2 - Final.docx

# An ecosystem for producing and sharing metadata within the web of FAIR Data

Daniel Jacob[1,6†], François Ehrenmann[2], Romain David[3], Joseph Tran[4], Cathleen Mirande-Ney[5], Philippe Chaumeil[2]

[†] Corresponding author

Institutional addresses:

[1] INRAE, Université de Bordeaux, UMR BFP, 71 av E Bourlaux, F-33140 Villenave d'Ornon, France

[2] INRAE, Université de Bordeaux, UMR BIOGECO, 69 route d'Arcachon, F-33610 Cestas, France

[3] European Research Infrastructure on Highly Pathogenic Agents (ERINHA AISBL), 98 rue du Trône B-1050 Bruxelles, Belgium

[4] INRAE, Université de Bordeaux, Bordeaux Sciences Agro, ISVV, UMR EGFV, F-33140 Villenave d'Ornon, France

[5] Université de Bordeaux, INRAE, UMR BFP, 71 av E Bourlaux, F-33140 Villenave d'Ornon, France

Present address:

[6] INRAE, UR1268 BIA, Centre INRAE Pays de Loire - Nantes, F-44000, Nantes, France

Email addresses, ORCID:

DJ: daniel.jacob@inrae.fr, ORCID: 0000-0002-6687-7169

FE: francois.ehrenmann@inrae.fr, ORCID:0000-0003-2727-0070

RD: romain.david@erinha.eu, ORCID: 0000-0003-4073-7456

JT: joseph.tran@inrae.fr, ORCID: 0000-0002-4624-0363

CMN: cathleen.mirande-ney@inrae.fr, ORCID: 0000-0001-9760-8482

PC: philippe.chaumeil@inrae.fr

## Abstract

**Background:** Descriptive metadata is vital for reporting, discovering, leveraging, and mobilising research datasets. However, resolving metadata issues as part of a data management plan can be **complex** for data producers. To organise and document data, various descriptive metadata must be created. Furthermore, when sharing data, it is important to ensure metadata interoperability in line with FAIR principles. Given the practical nature of these challenges, there is a need for management tools that can assist data managers effectively. Additionally, these tools should meet the needs of data producers and be user-friendly, requiring minimal training.

**Results**: We developed Maggot (Metadata Aggregation on Data Storage), a web-based tool to locally manage a data catalogue using high-level metadata. The main goal was to facilitate easy data dissemination and deposition in data repositories. With Maggot, users can easily generate and attach high-level metadata to datasets, allowing for seamless sharing in a collaborative environment. This approach aligns with many data management plans as it effectively addresses

challenges related to data organisation, documentation, storage, and the sharing of metadata based on FAIR principles within and beyond the collaborative group. Furthermore, Maggot enables metadata crosswalks, i.e., generated metadata can be converted to the schema used by a specific data repository or be exported using a format suitable for data collection by third-party applications.

**Conclusion**: The primary purpose of Maggot is to streamline the collection of high-level metadata using carefully chosen schemas and standards. Additionally, it simplifies data accessibility via metadata, typically a requirement for publicly funded projects. As a result, Maggot can be utilised to promote effective local management with the goal of facilitating data sharing while adhering to the FAIR principles. Furthermore, it can contribute to the preparation of the future EOSC FAIR Web of Data within the European Open Science Cloud framework.

## Background

In scientific research, metadata plays a crucial yet often overlooked role. Despite being essential for the discovery, reporting, and mobilisation of research datasets, metadata remains poorly understood in scientific communities. However, since metadata is data itself, it must be managed with the same level of rigor as the research data produced and consumed by research processes. This lack of awareness persists even in an era where sharing research data has become the cornerstone of open science initiatives and reproducible science. As transparency and collaboration become increasingly important to the scientific process, understanding the importance of metadata becomes imperative [**1**,**2**].

However, the production of metadata requires effort and expertise, and data producers and curators may be reluctant to make this additional time investment unless they see a tangible return [**3**]. Therefore, proactive approaches are needed to overcome this hurdle and educate data producers about the benefits of open data practices [**4**].

Furthermore, the creation of metadata poses challenges for data producers. Data management plans (DMPs) that describe strategies for managing research data throughout their life cycle often ask non-trivial questions. For example, they may inquire about the interoperability of data or about the type of metadata schema used. These questions can be difficult to answer, especially when datasets span various scientific domains and require input from people with varying skills [**5**]. The great diversity of research data and the wide variety of characteristics they describe further complicate metadata management [**6**].

Given the complexity of the issue, it is important to differentiate between the different types and functions of metadata. To keep things simple, we can divide metadata into two main groups: high-level and specialised metadata. The latter comprises structural metadata that describe the organisation and interconnections within a dataset. For example, structural metadata is essential to optimise the reuse of experimental data tables [**7**]. In contrast, high-level metadata (descriptive, administrative, rights) apply to all types of data generated within similar experimental contexts.

These two types of metadata can be considered either within the same data warehouse or separately. In the first case, the warehouse must be able to accommodate the data along with all associated metadata, typically resulting in repositories that are highly specialised (e.g., MetaboLights, https://www.ebi.ac.uk/metabolights/). In the second case, the different types of data produced can be distributed across various warehouses. Complementing successful initiatives describing experimental data arrays with ODAM [**7**], complex experiments with CEDAR [**8**] or omics data with the ISA-Tools suite [**9**], there are generalist data repositories such as Zenodo (https://zenodo.org/) or repositories based on the Harvard Dataverse software [**10**] (https://dataverse.harvard.edu) allowing users to deposit both high-level metadata and data that is not supported or insufficiently represented in existing data repositories.

High upstream in the metadata creation and curation chain, i.e., well before any dissemination, data - in all its diversity - needs to be managed locally. Even within the same project, the production of data can be spread out over several years and involve several partners and numerous individuals, including fixed-term staff such as doctoral students and postdoctoral fellows. In this situation, data management practices must provide transparency and ensure access to the collective's data assets, while adopting best practices such as the FAIR principles (Findable, Accessible, Interoperable, Reusable) [**11**].

Therefore, high-level metadata, a requirement for later data dissemination, is also highly relevant during the initial local data management. This should motivate data producers to provide a high level of documentation of their data, especially once they have been made aware of its benefits and the fact that this type of data only needs to be created once. In this work, we focused on the use of high-level metadata to locally manage a data catalogue, with the prospect of later being able to distribute the data more easily in a data repository. Our approach is based on the Maggot (Metadata Aggregation on Data Storage) software, specifically designed to facilitate the documentation of datasets using high-level metadata in the form of files that can be attached to the storage space.

## Design Considerations

Maggot (Metadata Aggregation on Data Storage) was developed to meet the need for a versatile data management tool that can support diverse annotation requirements. Its main objectives are to provide visibility of a collective's data assets, enable the general description of data, and promote the early adoption of FAIR principles. Furthermore, it ensures that data are kept in a format that is sustainable and facilitates reusability, particularly if data is produced by fixed-term staff (doctoral students and postdoctoral fellows), as it helps to create an awareness among less experienced staff members of the importance of good data description practices, thus fostering a culture of excellence in data management [**3**].

The wide range of scientific data is often managed separately using dedicated tools or repositories (omics data, experimental data tables, images, etc.). While each data type typically requires highly

specific structural metadata, it may be possible to define a common set of high-level metadata descriptors (i.e., descriptive, administrative, rights) that apply across a wide variety of data types and usage scenarios, including contexts that require collective data sharing. To address the challenge of managing all data, Maggot was developed by relying primarily on high-level metadata.

Data management plans (DMP) usually call for a centralised approach to data storage to ensure data safety (backup) and security (controlling access), i.e., outside of users' disk space, which becomes particularly important when fixed-term staff are involved in the data production. However, data managers must consider how such centralised storage spaces can be organised, e.g., through harmonising folder and file naming conventions and the use of README files to provide relevant information. The only constraint imposed by using Maggot is that each dataset must be associated with one root directory regardless of whether this contains the entire dataset or just a subset. If some data are stored elsewhere, they could be referenced using a URL for example. Regarding directory trees, they can be created in any fashion that suits data producers e.g., by project, theme, or team, or using a combination of these. Instead of (or in addition to) a README file, a high-level metadata file is deposited in each dataset directory to clearly identify the particular dataset. These high-level metadata files allow Maggot to create a data catalogue directly from the data repository. Maggot software has been specifically designed to provide effective answers regarding the choice, format and means of creating relevant high-level metadata.

While some tools (e.g., CEDAR [**8**], FAIRDOM-SEEK [**12**]) use both high-level and structural metadata descriptors, Maggot relies on high-level metadata only because they apply to all the data, while encouraging us to rely on other tools or online platforms specialised in a particular area or data type. For high-level metadata, the metadata schema should be chosen with some degree of foresight based on the data repository where the final data is to be deposited. In France, the national data repository (https://entrepot.recherche.data.gouv.fr) uses the Harvard Dataverse software, which is largely based on the standard DDI (Data Documentation Initiative) metadata schema (https://ddialliance.org). The advantage of the DDI schema is that it encompasses a wealth of background information that can describe data sets of any type. It is also more extensive than the DataCite (https://schema.datacite.org/) or DublinCore (https://www.dublincore.org/schemas/) schemas. Due to these advantages, Maggot uses the DDI schema by default, although it is possible to employ one of the other schemas mentioned.

Typically, the metadata schema implemented by generalist data repositories only offers a small set of metadata, which may serve as a starting point. Consequently, it will be necessary to add other pertinent metadata to facilitate the local data management. As the choice of high-level metadata is an important step, collaboration between the data manager and data producers is essential to agree on an adequate minimum set of metadata. Although challenging, it is crucial to meticulously build and adapt the schema to align with existing and future data needs [**13**].

The high-level metadata span several types of information to be documented (descriptive, administrative, rights) implying different types of terminology. Since standardisation is key to

interoperability, metadata must meet established standards and be described using controlled vocabulary widely accepted by the scientific community [**14**]. Hence, both the metadata and sources of vocabulary (ontologies, thesauri, dictionaries) must be agreed upon by data managers and producers. On the one hand, choosing metadata involves primarily the data producers who have direct knowledge of the data. This may represent a challenge because data producers may not be familiar with metadata standards and best practices. On the other hand, since data managers and data stewards have a high level of expertise in the application of FAIR principles [**11**] and metadata standards, they can play an important role in guiding the choice and management of metadata. In recognition of the complementarity of these roles, collaborative partnerships between data managers and scientists are essential to ensure effective and sustainable management of research data [**15**].

Data managers must raise awareness and encourage data producers to improve the quality and reusability of their data without requiring them to become subject matter experts [**3**]. This guidance is therefore only intended to provide recommendations on relevant metadata and controlled vocabulary for the relevant scientific area, as well as training data producers on best practices such as the use of permanent identifiers like DOI, ORCID, RoR, and other systems. Additionally, data producers should be informed about selecting appropriate licences such as CC-BY (https://creativecommons.org), data policies (FAIRSharing https://fairsharing.org/), or data repositories (Re3data, https://www.re3data.org/).

## Results

Maggot was designed for maximum usability, which meant keeping the user interface simple and automating metadata entry based on auto-completion whenever possible. However, the configuration remains slightly more complex, consisting of two configuration levels and several configuration tables in a spreadsheet (**Fig. 1**) (https://inrae.github.io/pgd-mmdt/definitions/).

### Metadata definition

At configuration level 1, the high-level metadata must be defined, a crucial step that will affect all future data management. The input and search interfaces are entirely generated based on the terminology file, which defines each field, the corresponding input type, and associated vocabulary. Another level 1 configuration file serves to document each term through examples and links to additional information if necessary. This provides contextual assistance accessible during data entry, guiding data producers as they fill in each form field. A complete example is provided (**Additional File 1**) and a detailed documentation of the set-up of configuration files is available online (https://inrae.github.io/pgd-mmdt/configuration/).

As mentioned above, Maggot uses the Harvard Dataverse metadata standards (DDI-based) by default, serving as a useful starting point from which users can make customizations. Because other choices are possible, users should consult the MIT online metadata documentation before making any changes to the schema (https://libraries.mit.edu/data-

management/store/documentation/). Furthermore, although metadata schemas should be linked to the FAIR principles [**16**], data producers should be able to modify any chosen schema if necessary to adequately describe the data. Maggot allows users to divide metadata into several sections, each section constituting a tab in the interface. Hence a section dedicated to specific metadata not included in the standard metadata schema could be created to facilitate efficient searches in the catalogue adapted to the given data type. The choice to extend the original metadata schema largely depends on the scientific field and the criteria that each collective wishes to establish for an adequate general description. Maggot possesses the necessary scalability and flexibility to allow the creation of high-level metadata tailored to any experimental context.

To ensure the effective management of controlled vocabularies, Maggot provides users with a choice of dictionaries and ontologies and allows them to create their own custom dictionaries (https://inrae.github.io/pgd-mmdt/dictionaries/). As it is unlikely that users will be able to conceive a complete set of appropriate terminologies right from the outset of a project, Maggot allows for an iterative and progressive approach. For example, users can start out with a simple dictionary based on local sources. As they start to consolidate their vocabulary, they can create a thesaurus (or a controlled vocabulary) that is separate from existing ontologies. To facilitate a quick start, Maggot can query thesauri directly from the SKOSMOS web application [**17**] (https://skosmos.org). In addition, ontologies can be chosen gradually as data producers gain a better understanding of the relevant terminology and usage context. Indeed, Maggot allows users to enrich their metadata using ontologies publicly accessible via OntoPortal web applications such as BioPortal [**18**] (https://bioportal.bioontology.org) and AgroPortal [**19**] (https://agroportal.lirmm.fr), but also via the EMBL-EBI Ontology Lookup Service (https://www.ebi.ac.uk/ols4).

**Metadata crosswalks**

At configuration level 2, users need to establish definitions of how their schema can be mapped onto a differently structured metadata format. This is termed “metadata crosswalk”. The DDI metadata schema that is used by default in Maggot is sufficiently rich to allow for mapping to other schemas such as DublinCore, for example. Maggot allows users to transform high-level metadata for deposition in data repositories. This can either be the default repositories that also use Maggot’s native DDI format such as Dataverse, or high-level metadata can be exported to other formats suitable for data harvesting (e.g. XML, JSON-LD) by third-party applications via an application programming interface (API), e.g., OAI-PMH (Open Archives Initiative Protocol for Metadata Harvesting - https://www.openarchives.org). These functionalities use a metadata crosswalk approach (https://inrae.github.io/pgd-mmdt/chats/chat4/) based on the mapping files defined at configuration level 2. These files map the metadata defined at level 1 onto the output metadata schema (**Fig. 1**). This ensures maximum compatibility with other systems, in line with FAIR principles. Furthermore, this approach ensures long term data preservation and facilitates a potential future migration away from Maggot. Maggot allows organisations to improve their data management practices, guaranteeing effective metadata use throughout its lifespan while facilitating data dissemination. Maggot also improves the interoperability and reusability of data

while increasing the possibilities for data coupling, as envisaged by international consortia such as the European Open Science Cloud (EOSC, https://open-science-cloud.ec.europa.eu).

## Features

Maggot's functionalities can be divided into three parts: creation, sharing, and distribution (**Fig. 2**).

High-level metadata capture can be initiated from the very start of a project and does not require all data to be available or processed. In fact, metadata can be added in an iterative fashion throughout the duration of the project. Maggot supports descriptive and administrative metadata for any type of data, relying on user-defined custom fields where necessary. The metadata entry occurs via a form (**Fig. 3**), which is auto-generated based on the level 1 configuration. A minimum set of fields are mandatory to ensure compatibility for later data deposits. The selection of mandatory fields can be changed at any time by the data manager, who also defines the data policy and its implementation and governance. In contrast, data stewards are responsible for the data quality and curation. Before depositing the high-level metadata file in the storage space, Maggot allows users to send the metadata file to the data stewards for validation purposes and quality control. Data stewards can also handle the final submission if the designated storage space has limited write access. For each metadata input field, a help dialogue is available to provide a definition of the field and instructions for completing it. It is the responsibility of data managers to maintain high quality documentation for data entry based on project requirements and users/data stewards feedback.

The sharing of data relies on both storage space and high-level metadata. To establish the search criteria, a form that closely resembles the entry form is provided (see Fig. 4). The quality of this research is dependent on the high-level metadata, which plays a crucial role at two levels. Firstly, it is important to carefully choose relevant metadata in order to effectively target a specific set of data from the storage space. Secondly, attention must be given to the data entry process and any subsequent curation. Therefore, it is recommended to minimise the use of open fields for free text entry. Whenever possible and appropriate, a controlled vocabulary should be used and enforced. However, since it is impossible to predict all scenarios in advance, Maggot allows for open fields for data entry over a long period of time. Any new entries can be regulated either by the data stewards through additions to dictionaries or by the system itself. In the latter case, Maggot provides fields where pre-recorded options can be selected, while still allowing users to enter new entries. These new entries are immediately recorded in the system and can then be proposed for selection to other users or datasets (https://inrae.github.io/pgd-mmdt/definitions/vocabulary/).
Sharing metadata does not imply sharing data. However, there are various ways to provide access to the data themselves before their distribution. One option is to install a file browser, either with or without passwords (https://inrae.github.io/pgd-mmdt/installation/). Another possibility is to deposit all the data in the collective's datacenter and include a link to this resource in the metadata file. Maggot allows for data fragmentation, meaning that data can be dispersed across different platforms, databases, and file formats. This allows data producers to specify resources, both external and internal, and centralise all links to the data (**Fig. 2**). External

resources should be specified using a URL, with a preference for a permanent identifier such as a DOI. Any URL that points to data and respects the FAIR principle can also be used. Additionally, in cases where local data management is applicable, it is advisable to indicate the location of the data if it is different from that of the metadata (e.g., NAS unit or data cloud). By bringing together all references to multiple data sources in one place, Maggot can function as a data hub.

The distribution of data requires high-level metadata. It is crucial to have the mapping file properly configured upstream so that metadata can be crosswalked from the original internal schema to the schema of the target repository, which may not use all metadata fields originally defined. Although Maggot is currently limited to two repository platforms (Dataverse and Zenodo), there may be support for others in the future (e.g., Dryad [**20**] and RO-Crate [**20**]). This also does not prevent the reuse of metadata. It is entirely possible, for instance, to establish an internal metadata harvesting process to automatically populate another data source, such as the FAIRDOM-SEEK data management platform [**12**]. By choosing Maggot, users are not restricted to this system as they can export the generated metadata to other formats and platforms, which ensures that future applications/services can still make use of legacy metadata thereby avoiding data loss. Maggot facilitates this by allowing data scientists and data repositories to harvest data. Through the OAI-PMH protocol users can retrieve all datasets based on the DublinCore schema, while the metadata can be collected in JSON-LD format (JSON for Linking Data, https://json-ld.org), which adheres to the schema.org standard (https://schema.org). This aspect is particularly critical for linking metadata in the linked data domain and ensuring interoperability. Future releases of Maggot will support DCAT-based harvesting (https://www.w3.org/TR/vocab-dcat-3/).

High-level metadata alone is insufficient to fully describe a dataset, and structural metadata is needed as well. For instance, when dealing with experimental data tables that are managed using ODAM [**7**], the structural metadata is provided in the "Frictionless data package" standard format (https://frictionlessdata.io/), which enables data users to easily parse the data. As a result, this datapackage file can be deposited in a data repository along with the high-level metadata (e.g., https://doi.org/10.15454/95JUTK). It is important to note that Maggot only handles high-level metadata for ODAM resources. Knowing that with ODAM data management also relies on storage space, perfect complementarity exists between these two tools, each managing a specific level of metadata.

Another usage scenario for Maggot is the production of high-level metadata that is directly pushed to a data repository without requiring a local storage. In this case, the dataset is not registered in the local data catalogue. This allows users to utilise the Maggot web interface thus benefiting from all its contributions facilitating the entry of metadata (including dictionaries, controlled vocabulary, etc.) instead of the web interface provided by the data repository.

## Implementation and Documentation

Deploying Maggot requires two infrastructure components: (1) a server to host the web application and (2) a data storage space. The server must be capable of running a Linux-based operating system and support containerization using Docker. The latter facilitates easy installation and administration The data storage can be local (e.g., NAS unit) or remote (e.g., cloud based). Data access can be managed via the rclone tool (https://rclone.org).

Maggot is a web-based PHP application that uses MongoDB (https://www.mongodb.com) to index all metadata obtained by scanning the disk storage at 30-minute intervals. In addition, Maggot utilises several remote vocabularies (thesauri and ontologies) that it queries via API to facilitate real-time imports, reducing the need to manually update information. For example, Maggot uses Twitter's Typeahead library (https://twitter.github.io/typeahead.js/) which allows data managers to easily implement a new vocabulary. The SKOSMOS thesauri and EMBL-EBI Ontology Lookup Service (OLS) have also been implemented in this way. While API access to vocabularies uses a caching mechanism to speed up the export of metadata to other formats (e.g., JSON-LD) or to push to a data repository (Dataverse, Zenodo), this caching mechanism is disabled when searching for a term by auto-completion in the input interface. As Maggot allows different vocabulary sources (e.g., BioPortal and EBI OLS), it is possible for two versions of the same ontologies to coexist. Hence, the list of ontologies for each source and field needs to be specified with care to prevent them from overlapping.

Additional documentation is available at https://inrae.github.io/pgd-mmdt/ and within the application itself, with detailed explanations of how the terminology should be constructed using associated vocabularies.

## Conclusion and perspectives

Maggot is a tool designed specifically for annotating datasets by generating high-level metadata files that can be linked to storage spaces. It addresses challenges related to data organization, documentation, storage, and sharing of metadata in line with FAIR principles. By covering as much of the research data lifecycle as possible, Maggot ensures efficient and sustainable management of research data and simplifies the adoption of FAIR principles. This enables organisations to increase the value and accessibility of their data assets. Additionally, Maggot's ability to disseminate metadata based on standard (machine-readable) schemas make it an important tool for the creation of the future EOSC FAIR Data Web, part of the European Open Science Cloud.
To date, Maggot is primarily used on the intranet of organisations and research units for processing and managing different types of data and metadata. Furthermore, efforts to build a community around this tool are underway (e.g., discussion blog, provision of several configurations for different areas of application). This is intended to help users with the installation, configuration, and use of Maggot. Future releases will allow exports in RO-Crate [**21**] format and metadata harvesting based on DCAT. Additionally, there are plans to implement centralised authentication mechanisms (SSO) to cater to the requests of multi-site institutions such as universities.

## Availability of Source Code and Requirements

- Project name: Maggot
- Project homepage: https://pmb-bordeaux.fr/maggot/
- Project code repository: https://github.com/inrae/pgd-mmdt
- Documentation: https://inrae.github.io/pgd-mmdt/
- Operating system(s): Platform independent
- Programming languages: PHP, python, JavaScript
- Licence: GNU GPL v3
- Maggot, RRID: SCR_025261
- Biotools: https://bio.tools/maggot

## Abbreviations

API: Application Programming Interface; DDI: Document, Discover and Interoperate; DMP: Data Management Plan; EOSC: European Open Science Cloud; FAIR: Findable, Accessible, Interoperable, Reusable; JSON: JavaScript Object Notation; JSON-LD: JSON for Linking Data; OAI-PMH: Open Archives Initiative Protocol for Metadata Harvesting; NAS: Network Attached Storage;

## Competing interest

The authors declare that they have no competing interests.

## Funding

D.J. was partly supported by the MetaboHUB project funded by the French National Research Agency (ANR-11-INBS-0010) and by the WAPNMR project funded by the French National Research Agency (ANR-21-CE21-0014). D.J., F.E., C.M.N. and J.T. were partly supported by the Bordeaux Plant Science (BPS) project funded by the Université de Bordeaux. D.J., F.E, C.M.N, J.T and P.C were partly supported by the French National Research Institute for Agriculture, Food and the Environment (INRAE). R.D. was supported by the European Research Infrastructure on Highly Pathogenic Agents (ERINHA AISBL).

## Authors' Contributions

Conceptualization: D.J, F.E, PC.; funding acquisition: D.J, F.E.; methodology: D.J., F.E, R.D.; software: D.J, F.E.; writing—original draft: D.J., R.D.; writing—review and editing: All authors. All authors read and approved the final manuscript.

## Acknowledgments

We thank Dr Catherine Deborde (INRAE UR BIA & PROBE Research Infrastructure, BIBS Facility Nantes) and Dr Annick Moing (INRAE UMR 1332 BFP, Bordeaux) for advice on the manuscript and for constructive reviews. We also thank Edouard Guitton (INRAE, Animal Health Department) for his fruitful feedback in the implementation of Maggot within his research infrastructure.

## Additional files

**Additional file 1** : Examples of metadata files along with corresponding definitions files within an Excel workbook.

## Figures

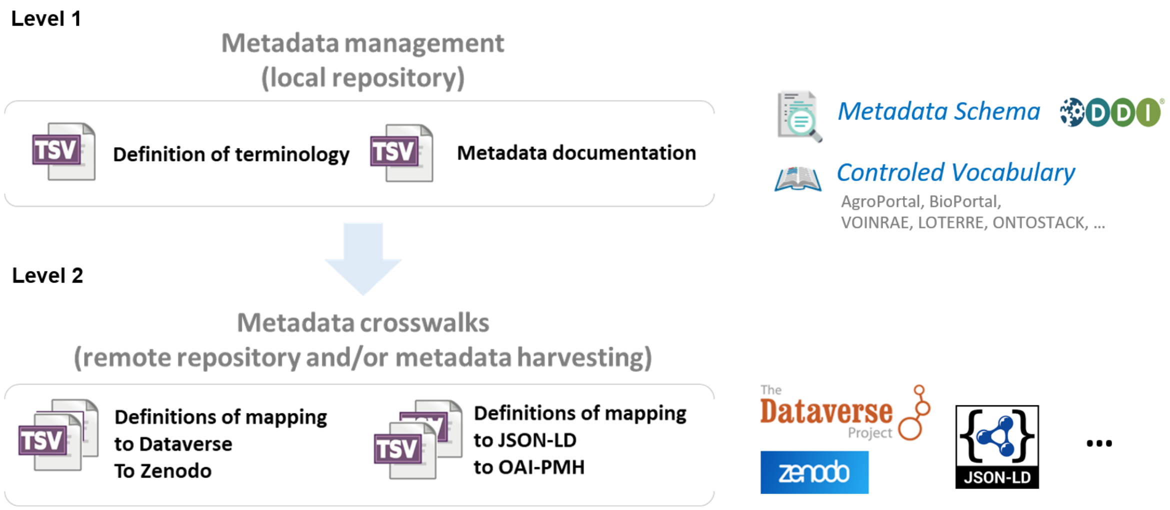


**Figure 1**: Maggot allows users to choose all the high-level metadata describing their data with two levels of definition files. The first level concerns the definition of metadata similar to a descriptive metadata plan. This category is more akin to configuration files, and constitutes the heart of the configuration around which everything else is based. The input and search interfaces are completely generated from these definition files, thus defining each of the fields, their input type and the associated controlled vocabulary. The second level concerns the definitions of the mapping to a differently structured metadata schema (metadata crosswalk, i.e., a specification for mapping one metadata standard to another), used either for metadata export to a remote repository (e.g., Dataverse, Zenodo) or for metadata harvesting (e.g., JSON-LD, OAI-PMH). The documentation gives the detailed workflow for the metadata dissemination in the Dataverse and Zenodo repositories showing the articulation of these two levels of configuration (https://inrae.github.io/pgd-mmdt/publish/).

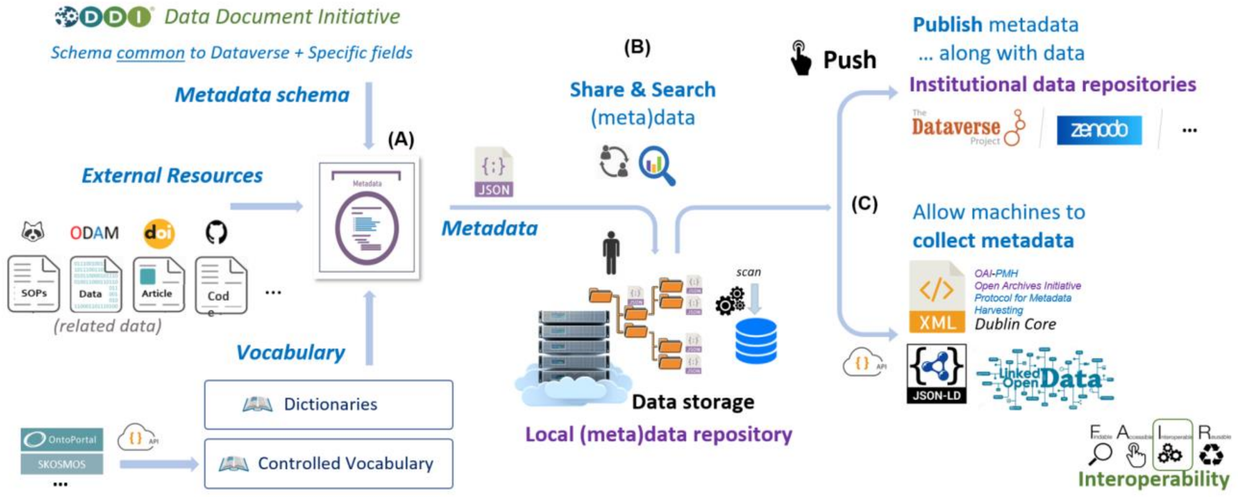


**Figure 2**: Main functionalities of Maggot split into three parts: creation, sharing and dissemination. (**A**) First, producing a document with metadata sets of data within a collective of people, thus allowing users to i) answer certain questions of the Data Management Plan (DMP) concerning data organisation, documentation, storage and sharing in the data storage space, ii) meet certain data and metadata requirements, listed for example by Open Research Europe in accordance with FAIR principles. (**B**) Next, searching for datasets by their metadata. Here, the descriptive metadata thus produced can be associated with the corresponding data directly in the storage space, making it possible to perform a search on the metadata to find one or more sets of data. Only high-level metadata is accessible by default. (**C**) Finally, publishing the high-level metadata of the datasets as well as their data files in a European-approved repository, with the possibility either to directly harvest the metadata via the OAI-PMH protocol, or to export the associated metadata with their semantic context for full interoperability.

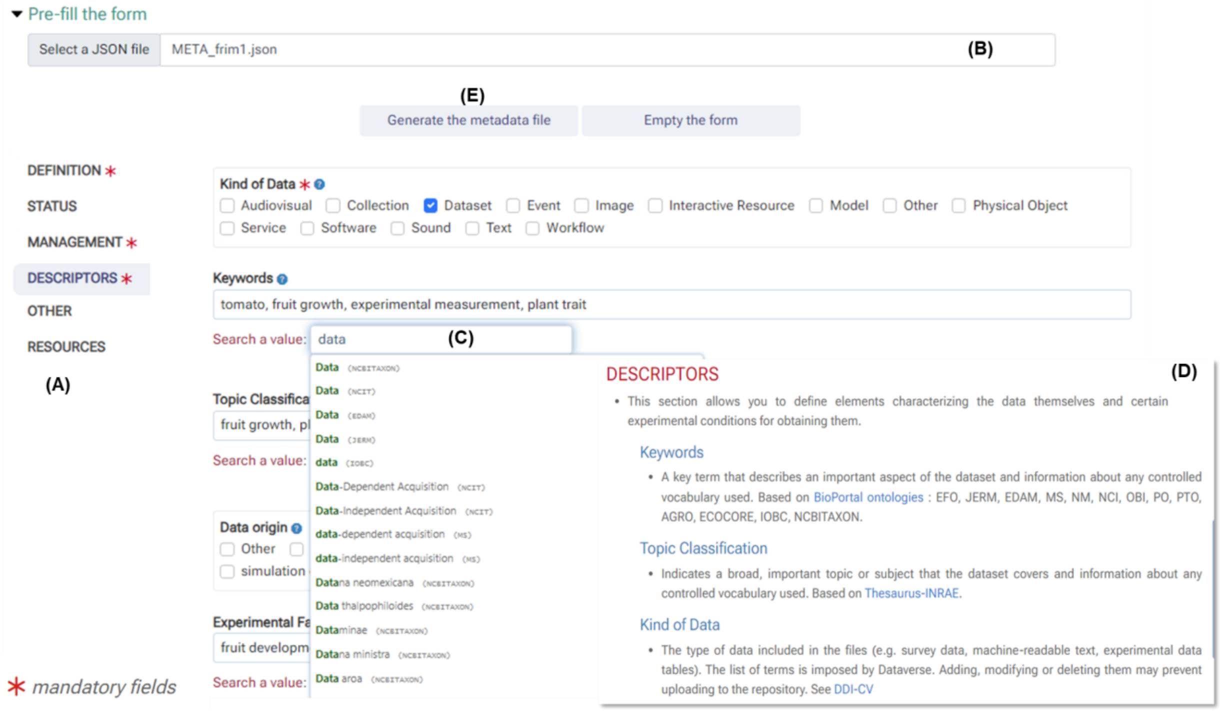

**Figure 3**: The metadata input form is generated based on the terminology file created during the initial system set-up. (**A**) Metadata fields are distributed between several tabs where related input fields are grouped together. (**B**) By loading a previously created metadata file, all form fields will be initialised to the predefined values. Mandatory fields are marked with a red asterisk. (**C**) Controlled vocabulary can be entered as free-form text, although the system offers to autocomplete entries based on a list of terms retrieved from the web. (**D**) Help for each input field can be obtained by clicking on a "?" icon, providing users with a definition of the corresponding field. (**E**) Once completed, the form can be saved as a file (an example has been provided as **Additional file 1**).

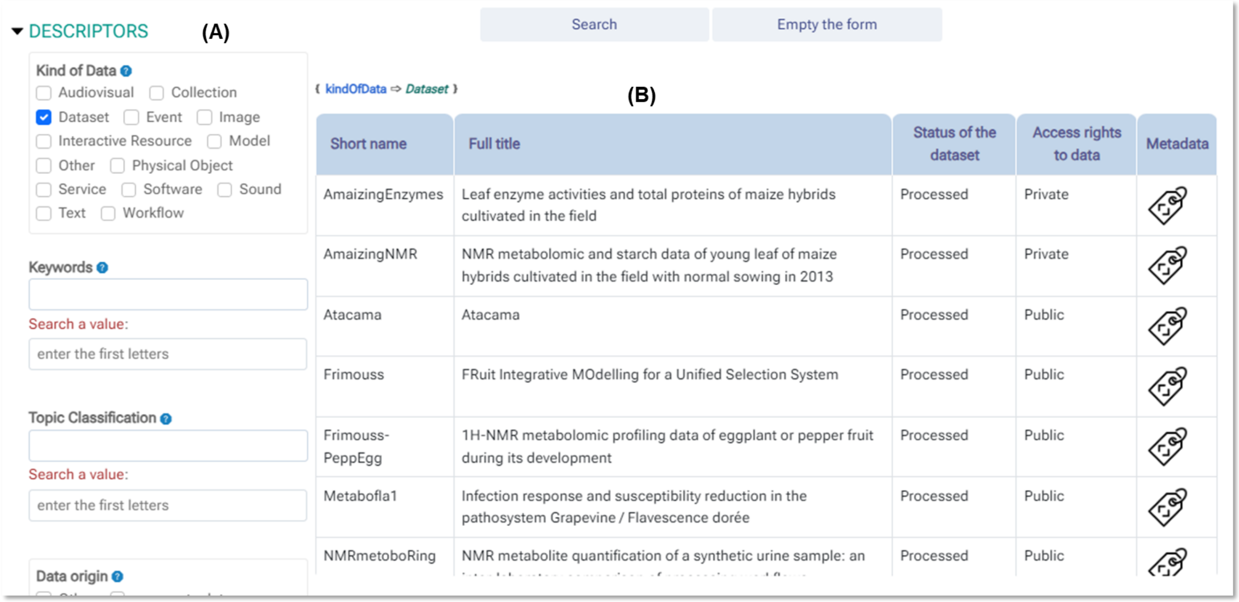


**Figure 4**: Maggot allows users to manage a data catalogue in the designated local storage space. The dataset search is divided into two parts: (**A**) a form almost identical to the entry form is provided to establish the search criteria using all user-provided metadata and (**B**) a table containing the default data sets. Only datasets meeting the search criteria will remain. Clicking on each of the columns in this table displays the data sets sorted accordingly. The column headers can be customised in the terminology definition file (https://inrae.github.io/pgd-mmdt/definitions/terminology/).

## References


1. Ulrich H, Kock-Schoppenhauer A, Deppenwiese N, et al. Understanding the Nature of Metadata: Systematic Review. J Med Internet Res. 2022;24(1):e25440. https://doi.org/10.2196/25440

2. Manninen L. Describing Data: A Review of Metadata for Datasets in the Digital Commons Institutional Repository Platform: Problems and Recommendations. J Libr Metadata. 2018. https://doi.org/10.1080/19386389.2018.1454379

3. David R, Mabile L, Specht A, et al. FAIRness Literacy: The Achilles' Heel of Applying FAIR Principles. CODATA Data Sci J. 2020. https://doi.org/10.5334/dsj-2020-032

4. Popkin G. Data sharing and how it can benefit your scientific career. Nature 2019;569:445-447. https://doi.org/10.1038/d41586-019-01506-x

5. David R, Baumann K, LeFranc Y, et al. Converging on a Semantic Interoperability Framework for the European Data Space for Science, Research and Innovation (EOSC), 2nd Workshop on Ontologies for FAIR and FAIR Ontologies. Sherbrooke, Québec (Canada). 2023a. https://doi.org/10.5281/zenodo.8102786

6. Çaldağ MT, Gökalp E. Understanding barriers affecting the adoption and usage of open access data in the context of organisations. Data Inform Manag. 2023. https://doi.org/10.1016/j.dim.2023.100049

7. Jacob D, David R, Aubin S, Gibon Y. Making experimental data tables in the life sciences more FAIR: a pragmatic approach. GigaScience 2020;9(12):giaa144. https://doi.org/10.1093/gigascience/giaa144

8. Musen M.A., Bean C.A., Cheung K.H., et al. The center for expanded data annotation and retrieval. J. Am. Med. Inform. Assoc. 2015;22:1148–1152. https://doi.org/10.1093/jamia/ocv048

9. Sansone S.A, Rocca-Serra P., Field D., et al. Toward interoperable bioscience data. Nature Genetics 2012, https://doi.org/10.1038/ng.1054

10. King G. An Introduction to the Dataverse Network as an Infrastructure for Data Sharing. Sociol Methods Res. 2007;36(2):173-199. https://doi.org/10.1177/0049124107306660

11. Wilkinson MD, Dumontier M, Aalbersberg IJJ, et al. The FAIR Guiding Principles for scientific data management and stewardship. Sci Data 2016;3:160018. http://dx.doi.org/10.1038/sdata.2016.18

12. Wolstencroft K, Owen S, Krebs O, et al. SEEK: A systems biology data and model management platform. BMC Syst Biol. 2015;9:33. http://doi.org/10.1186/s12918-015-0174-y

13. David R, Richard AS, Connellan C, et al. Umbrella Data Management Plans to integrate FAIR data: lessons from the ISIDORe and BY-COVID consortia for pandemic preparedness. Data Sci J. 2023b;22:35, pp. 1–15. https://doi.org/10.5334/dsj-2023-0350

14. Bingo S, Montenegro M. How to Create a Descriptive Metadata Plan. Sustainable Heritage Network. Accessed April 13, 2024. Sustainable Heritage Network

15. David R, Rybina A, Burel J-M, et al. "Be sustainable": EOSC-Life recommendations for implementation of FAIR principles in life science data handling. EMBO J. 2023c. e115008. https://doi.org/10.15252/embj.2023115008

16. Musen, M.A., O'Connor, M.J., Schultes, E. et al. Modeling community standards for metadata as templates makes data FAIR. Sci Data 9, 696 (2022). https://doi.org/10.1038/s41597-022-01815-3

17. Suominen O, Ylikotila H, Pessala S, et al. Publishing SKOS vocabularies with Skosmos. Manuscript submitted for review. June 2015. https://skosmos.org/publishing-skos-vocabularies-with-skosmos.pdf

18. Noy NF, Shah NH, Whetzel PL, et al. BioPortal: ontologies and integrated data resources at the click of a mouse. Nucleic Acids Res. 2009;37(suppl_2):W170–W173. https://doi.org/10.1093/nar/gkp440

19. Jonquet C, Toulet A, Arnaud E, et al. AgroPortal: A vocabulary and ontology repository for agronomy. Comput Electron Agric. 2018;144:126-143. https://doi.org/10.1016/j.compag.2017.10.012

20. Isard M, Budiu M, Yu Y, et al. Dryad: distributed data-parallel programs from sequential building blocks. ACM SIGOPS Oper Syst Rev. 2007;41(3). https://doi.org/10.1145/1272998.1273005

21. Soiland-Reyes S., Sefton P., Crosas M. et al. Packaging research artefacts with RO-Crate, Data Science 5(2) https://doi.org/10.3233/DS-210053

Click here to access/download
**Supplementary Material**
maggot_file_examples.xlsx

Personal Cover Click here to access/download;Personal Cover;Cover_letter.docx

Daniel Jacob
INRAE, Université de Bordeaux
71 av E Bourlaux
33140 Villenave d'Ornon, France
Tel. +33 557 571 228
daniel.jacob@inrae.fr

May 17, 2024

GigaScience,
Managing Editor

Dear Editor,

We are delighted to submit our manuscript entitled "Maggot: An ecosystem for sharing metadata within the web of FAIR Data" for consideration as a Technical Note in GigaScience. Our work introduces the Maggot tool, designed to enhance the production, sharing, and distribution of metadata within the scientific community.

Maggot serves as an essential bridge connecting data producers, responsible for data documentation and dissemination but not very experts in open data, with the data manager responsible for ensuring compliance with FAIR principles, the use of standard metadata schemas and of a controlled vocabulary recognized by the community. in the targeted scientific field. This tool therefore goes in the right direction for the construction of the FAIR data network, a key objective under the European Open Science Cloud (EOSC), aimed at facilitating more accessible and reusable research data across Europe.

We believe the Maggot tool addresses a pressing need and aligns closely with the aims and scope of your journal. We are eager for the opportunity to contribute to the discourse in your readership and are confident our tool will be of significant interest.

Please let us know if you need any additional information or details; we are prepared to respond promptly.

Thank you for considering our manuscript.

Sincerely,

Daniel Jacob

Research engineer in data science,

UMR 1332 BFP, INRAE, Université de Bordeaux
