# Supplementary material for: An ecosystem for producing and sharing metadata within the web of FAIR Data
Source: Gigascience. 2025 Jan 8;14:giae111. doi: 10.1093/gigascience/giae111 (PMC11707607; doi:10.1093/gigascience/giae111)
Supplement: giae111_Response_to_Reviewer_Comments_Original_Submission [file giae111_response_to_reviewer_comments_original_submission.pdf]

Author's Response To Reviewer Comments

Dear Editor,

Concerning manuscript " Maggot: An ecosystem for sharing metadata within the web of FAIR Data " (GIGA-D-24-00167), all remarks of reviewers have been considered.
The reviewers noted a lack of detail and clarity, with the manuscript appearing too general. This issue stemmed from our initial intent to write a longer and more comprehensive article. However, we ultimately decided to focus on a more technical piece. The version originally submitted unfortunately reflected this change in direction inadequately. In the revised version, we have fully embraced the technical focus, ensuring that any overly general arguments have been removed unless they explicitly support our approach.
Reviewers, experts in the field, provided us with valuable revision elements to improve the manuscript. We were keen to satisfy the various advice and remarks, especially since they were provided in a spirit of benevolence with a real interest for the content of the manuscript.
The answers and changes that have been made are detailed below.
Additionally, we submitted the revised manuscript to a native English speaker for a thorough language review.
Please let us know if some additional modifications are needed.
Best wishes,

Daniel Jacob

Reviewer reports:

Reviewer #1:

* The authors describe in very general terms a system to assist in the creation and management of experimental metadata. The paper provides a thoughtful description of how a metadata-management system needs to fit into an overall data ecosystem, tracing the flow of requirements from data-management plans to the archival of results in an online repository. The authors do not provide enough information about their system, however, for the reader to understand what has been accomplished. What does the system look like? Who has used it? What has the system done well? What have the problems been? What future work is planned to address the problems? In the absence of more information about the system—its architecture, its user interface, and its metadata model—and in the absence of any evaluation of the system, it's hard to know what the reader should take away.

Answer: We completely agree with your observations. As explained to the editor, our initial plan was to write a longer and more general article, but we ultimately decided to focus on a more technical piece. Unfortunately, the originally submitted version reflects this shift in direction poorly. In the revised and corrected version, we have fully embraced the choice of a technical article. However, in the initial version, we presented certain aspects inadequately, and the online documentation could have provided additional information.
In this revised version, we now fully explain our choices in the “Design Considerations” section, particularly regarding the metadata schema (lines 150-161). In the “Results” section, we now describe in detail the different stages of the configuration and provide recommendations concerning the choice of metadata, as well as an approach to the selection of controlled vocabularies. Furthermore, the three main functionalities are now described with two figures (Figures 3 and 4) used to illustrate them.

* There are open questions regarding general-purpose metadata models (such as the Dataverse model, the DataCite schema, DCAT, and DDI), to which the authors appear to be committed, and the relationship of these general frameworks to discipline-specific metadata standards (such as those available through Fairsharing.org). The FAIR Guiding Principles come down on the side of discipline-specific metadata

standards, and the authors would seem to be well positioned to contribute to dialog in this regard.

Answer: This is a very interesting topic indeed, and it was the subject of the initial article. However, in this technical article, we focus on our choice based more on practical reasons (national repository based on Dataverse / DDI) than a discussion on the good criteria for choosing a metadata schema. That said, since the Maggot software allows users to select their own schema, such a discussion could be the subject of a separate article. As noted in our revised conclusion, we want to create a community around this tool, and these questions will be for sure at the heart of our concerns.

* The paper surprisingly has no discussion of related work. An advantage of open review is that I, as a referee, do not need to feel sheepish in suggesting that my own work is probably the most relevant here. My laboratory has been working on a system called CEDAR for nearly ten years. CEDAR has been adopted by several large scientific consortia, and it is now integrated into the data/metadata-uploading facilities of generalist data repositories such as Dryad and OSF. I'd be happy to discuss CEDAR with the authors outside of this review. One can read more about our work here: https://www.nature.com/articles/s41597-022-01815-3

Answer: We did not consider it necessary to include a discussion section. However, in this revised version, we justify our choices in relation to other approaches (all types of metadata in a single data warehouse - e.g. CEDAR, FAIRDOM-SEEK, MetaboLights vs. metadata distributed across multiple repositories) in the "design considerations" section (lines 150-161).
Thank you for the reference which we read with interest and have suggested to our potential readers Indeed, the CEDAR system seems to us to be very relevant for the creation of metadata and data management in a spirit of open scientific data, following the FAIR principles. Although we have not been able to evaluate it in practice, our experience with numerous data producers suggests that the more exhaustive a system aims to be, the more reluctance there is to document the data. Unfortunately, many people prefer to deposit OMICS data directly into a general repository, even though there are more relevant specialised repositories available. This demonstrates that many of us are still far from fully embracing open data management in line with the FAIR principles. We emphasise at the start of the “Background” section the need to “reward the good actors” if we want this to progress.

* The authors may not be aware, but the word "maggot" has a rather unfortunate meaning in English.

Answer: It is a point of view. It becomes difficult to find a name that is a little original. So, this one stands out. Shouldn't the maggots be rehabilitated? ;-) Besides, in French, "un mago" is slang for a lucky find or a great discovery—so maybe we are just ahead of the curve on this one!

Reviewer #2:

* The reviewer thank the authors for their work, which presents a set of software components working together for the creation, editing and sharing of metadata. All code is made available via github code repository and is supported by extensive documentation.

Answer: We are very pleased that you highlight all our work. Thanks.

Main comments:

* A large amount of work and engineering went into the Maggot ecosystem. The communication could however be improved by clarifying the narrative.
The focus of the Maggot is 'generic metadata' reporting as opposed to domain specific metadata (e.g., metadata specific to report experimental details, instrumentation specifics, experimental results).

Answer: Indeed, it is one of the main focuses. In this revised article, we preferred the adjective “high-level” instead of generic, general, or even descriptive. This helps to better emphasise the fact that this metadata is like one of the “hats” on a dataset.

* In the 'Design Consideration' section, the authors highlight their structured approach starting with descriptor identification, terminology definition or adoption and finally standard selection.
Can the authors clarify the following sentence "Rather than mandating data conformity, the schema should be flexible to accommodate pre-existing data” in the second paragraph of that section?

Answer: This sentence means that it is more important to consider reality, in this case existing data, than standards with which we must willy-nilly deal. This sentence was a bit clumsy. It no longer appears in the revised version.

* In the following section about 'controlled terminologies', can Maggot be set to terminology services based on Bioportal? for instance, is it possible to interact with EMBL-EBI Ontology Lookup Service or to a custom service? What would be the technical requirements? It might be helpful to provide a pointer to Maggot documentation on that topic.

Answer: Ontologies based on OntoPortal (not only BioPortal, but also AgroPortal for example) are natively supported in Maggot. Additionally, we have implemented a mechanism based on Twitter's Typeahead library, allowing Maggot to support almost any controlled vocabulary accessible via API. For instance, the APIs for the SKOSMOS thesauri are supported in this way; they were managed specifically before but less well. Moreover, the EBI ontologies via OLS are also supported via this library. This approach allows for harmonising the use of various API-based vocabulary services, including OntoPortal APIs. An addition has been made to the online documentation regarding this and template scripts are provided with the source code for ease of implementation. See https://inrae.github.io/pgd-mmdt/definitions/vocabulary/
A test version is available online: (https://pmb-bordeaux.fr/maggot-vt/entry). In the “Descriptors” section, the CVs concerning the field “Topic Classification” are based on the thesaurus-inrae (SKOSMOS) and those concerning the fields “Experimental factor”, “Type of measurement” and “Type of technology” are based on OLS.

* In the next paragraph, as the authors argue for raising awareness in existing resources, pointing to resources such as FAIRSharing.org, Re3data or Bioregistry.org would be useful.

Answer: This paragraph has been shortened in the revised version, and placed at the end of the section. We have also added these resources (lines 188-191). Although, they are more useful to data managers and data stewards than to data producers.

* In the following section, can the authors specify provide an example of Core Trust seal repository have targeted ? Similarly, in the case of submitting metadata via API, which schema have need chosen or can be selected (e.g., datacite, dcat)?

Answer: We have removed this sentence related to the Core Trust seal repository in the revised version. No longer applicable.
Furthermore, in the case of submitting metadata via API, it is the metadata crosswalks definition file that defines the chosen schema compliant with the targeted repository. This is now better explained in the subsection "Metadata crosswalks definition" (lines 244-261).

* What is " a metadata crossing approach based on the mapping defined upstream by the data manager"? can it be simplified by saying that 'mappings are available between selected models"? How many models have been mapped and are available out of the box?

Answer: This sentence is not clear, indeed. So, we rewrote it: “These functionalities use a metadata crosswalk approach based on the mapping files defined at this second level. These mapping files map the metadata defined at the first level to the output metadata schema (Fig. 1)” (lines 252-255).

* Providing a pointer to the extensive maggot documentation would be a nice addition:
https://inrae.github.io/pgd-mmdt/chats/chat4/

Answer: Ok, good suggestion. Done (line 253)

* The last paragraph of the section seems to somehow contradict an earlier section claiming that "creating metadata should not compel data producers to engage in training in topics or concepts outside their expertise, such as FAIR principles, the semantic web, or metadata schema, unless they choose to do so" with the following sentence "Unavoidably, the development and dissemination of metadata, involving a metadata-centric culture, underline the need for ongoing training initiatives and data stewards are pivotal in educating data producers".

Answer: By "metadata-centric culture" we mean that users must be fully aware that without metadata, their data has little or no use. It is an inseparable whole. So every time we mention data it should also imply the metadata without having to specify it. On the other hand, users do not necessarily need to know concepts such as the notion of metadata schema, or even the FAIR principles. Not to mention the semantic web and other Link Data. In this revised version, we did not wish to develop this point, so only a small paragraph remains at the end of the "Design considerations" section (lines 184-191).

*Result section:

* s/supporting the wide data diversity range within a collective./ supporting a community's varied annotation needs/

Answer: Ok corrected (line 118).

* The first 2 sentences of the third paragraph to section may be at ease in the design consideration section, as it describes to decoupling a storage and metadata creation.

Answer: Ok we agree with this suggestion. Done (lines 134-148).

* The authors introduced Maggot as tool specifically targeting 'generic metadata'. However, in the following sentence, they state " Maggot supports the input of both descriptive and administrative metadata for any type of data, including datasets, images, sequences, and more, with customizable field definitions to suit diverse user requirements. Moreover, Maggot emphasises ease of use and adaptability." How can users know when they cross over into domain specific metadata?

Answer: In the revised version, we have removed this ambiguity by focusing only on high-level metadata - see the second paragraph of the "design considerations" section (lines 126-132). Also, there should be no question of metadata specific to a particular data type. It is nevertheless still possible to define such metadata, but we believe that this would be a misuse of Maggot.

* ODAM appears in figure 2. Can the authors provide more information about the relation with Maggot and ODAM when it comes to metadata description. Can Maggot be used to index metadata declared in ODAM or is maggot solely concerned with generic metadata about the ODAM resources (authors, owner, organization, terms of use)?

Answer: Good suggestion. We have added a paragraph at the end of this section explaining the connection between ODAM and Maggot (lines 328-336).

* In the fourth paragraph on the section, it could be beneficial to introduce the selection of OAI-PMH earlier and then explain the benefit it provides in terms of long term preservation and/or migration away from Maggot.

Answer: Ok. We added some words to this effect in the "Metadata crosswalks definition" paragraph (lines 248-257).

* Does Maggot support the export of metadata as schema.org based json-ld payloads natively?

Answer: Yes, it does. But on condition of having defined the mapping file ensuring the metadata crosswalk to the desired schema. In the mapping proposed by default, we base ourselves on schema.org. But it

would be entirely possible to choose another scheme like DublinCore.

*Implementation and Documentation section:

* Can the authors expand on the issue of access to controlled vocabularies via APIs? In particular, if any caching mechanism is in place and if different versions of specific vocabularies can be fetched or specified?

Answer: Ok. We have now clarified these points in this section (lines -359-365).

* The authors should be commanded for the extensive mkdocs based documentations. It may be nice to amend some of the figures (e.g., figure 3, https://inrae.github.io/pgd-mmdt/publish/) so they can highlight the availability of that excellent resource for the readership

Answer: Ok we agree with this suggestion. Done. The Fig.3 becomes now the Fig. 1 in the revised version.

*Conclusion Section:

* The first sentence feels a bit too long. break in 2 and push the «frictionless FAIR metadata sharing' to another sentence. the authors should be mindful of using 'frictionless' in this context due to use of frictionless data framework in ODAM, as a possible source of confusion. suggestion = s/frictionless/native/

Answer: OK the first sentence has been rewritten (lines 374-376).

* The last sentence of the first section leads to the following question: Does maggot allow granular specification of terms of uses (i.e, beyond the provision of a uri to a license document) ? If so, it would nice to add a figure or code snippet showing how this is done.

Answer: The point was indeed intended to be more general. To maintain focus on the scope of the tool, this sentence has been deleted. Maggot specifically allows you to specify conditions of use for data associated with high-level metadata. However, it doesn't prevent specific conditions of use from being specified for each type of data deposited in other data repositories.

Minor comments:

* Title: What is the "web of FAIR data"? could be the title be simplified to "Maggot : ecosystem for producing and sharing metadata to support FAIR data practice "?

Answer: The suggestion to add production in the title suits us completely. As for the "Web of FAIR data", we base ourselves on the objectives set by the EOSC: see https://beopen-project.eu/storage/files/parti-2-eosc-a-web-of-fair-data-k-luyben.pdf

* Abstract.background: order -> 1/reporting, 2/discovery, 3/mobilisation. then "poses challenges to data producers"

Answer: OK we agree with this suggestion. Done.

* s/" in alignment with FAIR principles/"in accordance to the FAIR principles"/

Answer: OK corrected

* Abstract.results: "Comprehensible" - some ambiguity here as to where it should be "comprehensive" or "explicit"? Then a question about "Collaborative environment" . Does it mean that Maggot allows concurrent editing of the same document by multiple users (as in a Google document for instance)?

Answer: Not really. We rewrite the sentence as : "With Maggot, users can easily generate and attach high-level metadata to datasets, allowing for seamless sharing in a collaborative environment." (lines 37-38).

* s/for enabling/ to enable/

Answer: OK corrected

Thank you for all the criticisms and suggestions which helped clarify the narrative. Very appreciated!
