# Supplementary material for: An ecosystem for producing and sharing metadata within the web of FAIR Data
Source: Gigascience. 2025 Jan 8;14:giae111. doi: 10.1093/gigascience/giae111 (PMC11707607; doi:10.1093/gigascience/giae111)
Supplement: giae111_Response_to_Reviewer_Comments_Revision_1 [file giae111_response_to_reviewer_comments_revision_1.pdf]

Author's Response To Reviewer Comments

Reviewer reports:

Reviewer #1: The paper is improved from the previous submission, and the authors have done a good job of responding to most of the previous comments. The paper now mentions related work, which is an important addition.

Q: There is still a problem in that the authors do not put their work in perspective and discuss why one might want to use their tool rather than some other system that might be available. It really is incumbent on the authors to provide this kind of information to a prospective user who would want to understand the advance that the authors have achieved.

A: As mentioned in the revised article (lines 149-152), Maggot only handles high-level or common metadata unlike many other tools, e.g. CEDAR. We added a few sentences to better emphasize this aspect :

“This therefore leaves open to other tools the description of the data themselves (specialized, structural metadata), which can be of a very great diversity. For example, an image management tool like OMERO (https://www.openmicroscopy.org/omero/) being dedicated to this type of data is therefore more able to describe them than a tool like Maggot. On the other hand, Maggot makes it possible to make the link between all the data. This approach allows metadata to be managed by mobilizing tools each dedicated to a particular type of metadata, thus leaving open the choice of possibilities. This approach is particularly advantageous for projects with a diversity of data types to process.”

Furthermore, lines 336-344, we give an example of complementarity between the Maggot and ODAM tools, each managing its corresponding level of metadata.

Q: This reviewer has difficulty with the terms "high-level metadata" and "specialized metadata." I appreciate the distinction that the authors are trying to make, but it is not obvious to the reader what makes metadata "high level" or "specialized." Metadata about the nature of the experiment (which the authors presumably would call "high-level"), for example, describe something rather "specialized." Terms such as "experiment-specific" and "file-specific" might convey the intended meaning more clearly.

A: There seems to be no standardized term yet that groups together descriptive, administrative and right metadata, while distinguishing it from metadata that describes the data itself and not its context. We could just as well have called it contextual metadata. Metadata about the nature of the experience, for example, is not about the data itself, and several types of data could potentially be generated with the same context.

Q: It is not clear why the authors state that, "for high-level metadata, the metadata schema should be chosen with some degree of foresight based on the repository where the final data will be deposited" (line 153). Given the authors' definition of "high-level metadata," these metadata indeed should be independent of the particular repository used. For example, high-level metadata authored using CEDAR can currently be uploaded to Dryad, OSF, and other generalist repositories without any schema-specific concerns. If a particular repository requires a particular schema, of course, then that is another matter.

A: Yes, in principle, we agree with this regarding metadata schemas that meet the community's expectations to make data "Findable" in particular. Provided that the chosen schema meets these expectations!
This is why a metadata schema close to that of the repository will then make mapping (or metadata

crosswalk) easier, i.e. establishing correspondences between the metadata created and those expected by the repository.It is worth noting that using Maggot could simply be to use its interface as an intermediary for entering metadata instead of the repository one (lines 346-350), benefiting from advantages such as dictionaries or ontologies, thus avoiding going through the repository interface which can be very tedious and time-consuming, for example, in entering authors and other contributors. Similarly, mandatory metadata: with a similar metadata schema, we can ensure that we have to enter this metadata.

Q: It unclear what it means for a metadata schema to be "linked" to the FAIR principles (line 217). The FAIR principles do maintain that metadata should be "rich" and that they should adhere to "community standards," something that very general schemas such as DDI do not support. When the authors discuss the extensibility of the metadata schemas provided by their tool, it would be helpful if they could clarify that this extensibility is needed to overcome the limitations of DDI and to enable data FAIRness in a way that is not supported by many proposed metadata standards.

A: It is worth noting that Maggot allows the use of any schema including a custom-created schema. Also, it is worth remembering the need to include in the schema the metadata expected by the community to make the metadata "Interoperable"; so metadata schema needs to be "linked” to the FAIR principles. Again, Maggot only handles high-level metadata. So a metadata schema like DDI fits the FAIR criteria perfectly.

Q: The title of the paper suggests that the authors are presenting an entire "ecosystem," although the manuscript describes only Maggot.

A: Maggot involves the use of external tools (e.g. thesaurus API, ontologies), and is inserted into an ecosystem (data repository, storage space, etc.). This is why we are talking about an ecosystem, without which Maggot would be nothing.
