# Supplementary material for: An ecosystem for producing and sharing metadata within the web of FAIR Data
Source: Gigascience. 2025 Jan 8;14:giae111. doi: 10.1093/gigascience/giae111 (PMC11707607; doi:10.1093/gigascience/giae111)
Supplement: giae111_Response_to_Reviewer_Comments_Revision_2 [file giae111_response_to_reviewer_comments_revision_2.pdf]

Author's Response To Reviewer Comments

Dear Editor,

Please find attached the modified manuscript as requested.

Kind regards

Daniel Jacob
