# Supplementary material for: An ecosystem for producing and sharing metadata within the web of FAIR Data
Source: Gigascience. 2025 Jan 8;14:giae111. doi: 10.1093/gigascience/giae111 (PMC11707607; doi:10.1093/gigascience/giae111)
Supplement: giae111_Reviewer_1_Report_Original_Submission — Mark Musen -- 6/10/2024 Reviewed [file giae111_reviewer_1_report_original_submission.pdf]

Reviewer Report

Title: An ecosystem for producing and sharing metadata within the web of FAIR Data

Version: Original Submission **Date:** 6/10/2024

Reviewer name: Mark Musen

Reviewer Comments to Author:

The authors describe in very general terms a system to assist in the creation and management of experimental metadata. The paper provides a thoughtful description of how a metadata-management system needs to fit into an overall data ecosystem, tracing the flow of requirements from data-management plans to the archival of results in an online repository. The authors do not provide enough information about their system, however, for the reader to understand what has been accomplished. What does the system look like? Who has used it? What has the system done well? What have the problems been? What future work is planned to address the problems? In the absence of more information about the systemâ€"its architecture, its user interface, and its metadata modelâ€"and in the absence of any evaluation of the system, it's hard to know what the reader should take away.There are open questions regarding general-purpose metadata models (such as the Dataverse model, the DataCite schema, DCAT, and DDI), to which the authors appear to be committed, and the relationship of these general frameworks to discipline-specific metadata standards (such as those available through Fairsharing.org). The FAIR Guiding Principles come down on the side of discipline-specific metadata standards, and the authors would seem to be well positioned to contribute to dialog in this regard.The paper surprisingly has no discussion of related work. An advantage of open review is that I, as a referee, do not need to feel sheepish in suggesting that my own work is probably the most relevant here. My laboratory has been working on a system called CEDAR for nearly ten years. CEDAR has been adopted by several large scientific consortia, and it is now integrated into the data/metadata-uploading facilities of generalist data repositories such as Dryad and OSF. I'd be happy to discuss CEDAR with the authors outside of this review. One can read more about our work here: https://www.nature.com/articles/s41597-022-01815-3The authors may not be aware, but the word "maggot" has a rather unfortunate meaning in English.

Level of Interest

Please indicate how interesting you found the manuscript: Choose an item.

Quality of Written English

Please indicate the quality of language in the manuscript: Choose an item.

Declaration of Competing Interests

Please complete a declaration of competing interests, considering the following questions:

- Have you in the past five years received reimbursements, fees, funding, or salary from an organisation that may in any way gain or lose financially from the publication of this manuscript, either now or in the future?
- Do you hold any stocks or shares in an organisation that may in any way gain or lose financially from the publication of this manuscript, either now or in the future?
- Do you hold or are you currently applying for any patents relating to the content of the manuscript?
- Have you received reimbursements, fees, funding, or salary from an organization that holds or has applied for patents relating to the content of the manuscript?
- Do you have any other financial competing interests?
- Do you have any non-financial competing interests in relation to this paper?

If you can answer no to all of the above, write 'I declare that I have no competing interests' below. If your reply is yes to any, please give details below.

I declare that I have no competing interests.

I agree to the open peer review policy of the journal. I understand that my name will be included on my report to the authors and, if the manuscript is accepted for publication, my named report including any attachments I upload will be posted on the website along with the authors' responses. I agree for my report to be made available under an Open Access Creative Commons CC-BY license (http://creativecommons.org/licenses/by/4.0/). I understand that any comments which I do not wish to be included in my named report can be included as confidential comments to the editors, which will not be published.

Choose an item.

To further support our reviewers, we have joined with Publons, where you can gain additional credit to further highlight your hard work (see: https://publons.com/journal/530/gigascience). On publication of this paper, your review will be automatically added to Publons, you can then choose whether or not to claim your Publons credit. I understand this statement.

Yes Choose an item.
