# Supplementary material for: An ecosystem for producing and sharing metadata within the web of FAIR Data
Source: Gigascience. 2025 Jan 8;14:giae111. doi: 10.1093/gigascience/giae111 (PMC11707607; doi:10.1093/gigascience/giae111)
Supplement: giae111_Reviewer_1_Report_Revision_1 — Mark Musen -- 9/22/2024 Reviewed [file giae111_reviewer_1_report_revision_1.pdf]

Reviewer Report

Title: An ecosystem for producing and sharing metadata within the web of FAIR Data

Version: Revision 1 **Date:** 9/22/2024

Reviewer name: Mark Musen

Reviewer Comments to Author:

The paper is improved from the previous submission, and the authors have done a good job of responding to most of the previous comments. The paper now mentions related work, which is an important addition. There is still a problem in that the authors do not put their work in perspective and discuss why one might want to use their tool rather than some other system that might be available. It really is incumbent on the authors to provide this kind of information to a prospective user who would want to understand the advance that the authors have achieved.This reviewer has difficulty with the terms "high-level metadata" and "specialized metadata." I appreciate the distinction that the authors are trying to make, but it is not obvious to the reader what makes metadata "high level" or "specialized." Metadata about the nature of the experiment (which the authors presumably would call "high-level"), for example, describe something rather "specialized." Terms such as "experiment-specific" and "file-specific" might convey the intended meaning more clearly.It is not clear why the authors state that, "for high-level metadata, the metadata schema should be chosen with some degree of foresight based on the repository where the final data will be deposited" (line 153). Given the authors' definition of "high-level metadata," these metadata indeed should be independent of the particular repository used. For example, high-level metadata authored using CEDAR can currently be uploaded to Dryad, OSF, and other generalist repositories without any schema-specific concerns. If a particular repository requires a particular schema, of course, then that is another matter.It unclear what it means for a metadata schema to be "linked" to the FAIR principles (line 217). The FAIR principles do maintain that metadata should be "rich" and that they should adhere to "community standards," something that very general schemas such as DDI do not support. When the authors discuss the extensibility of the metadata schemas provided by their tool, it would be helpful if they could clarify that this extensibility is needed to overcome the limitations of DDI and to enable data FAIRness in a way that is not supported by many proposed metadata standards.The title of the paper suggests that the authors are presenting an entire "ecosystem," although the manuscript describes only Maggot.

Level of Interest

Please indicate how interesting you found the manuscript: Choose an item.

Quality of Written English

Please indicate the quality of language in the manuscript: Choose an item.

Declaration of Competing Interests

Please complete a declaration of competing interests, considering the following questions:

- Have you in the past five years received reimbursements, fees, funding, or salary from an organisation that may in any way gain or lose financially from the publication of this manuscript, either now or in the future?
- Do you hold any stocks or shares in an organisation that may in any way gain or lose financially from the publication of this manuscript, either now or in the future?
- Do you hold or are you currently applying for any patents relating to the content of the manuscript?
- Have you received reimbursements, fees, funding, or salary from an organization that holds or has applied for patents relating to the content of the manuscript?
- Do you have any other financial competing interests?
- Do you have any non-financial competing interests in relation to this paper?

If you can answer no to all of the above, write 'I declare that I have no competing interests' below. If your reply is yes to any, please give details below.

I declare that I have no competing interests.

I agree to the open peer review policy of the journal. I understand that my name will be included on my report to the authors and, if the manuscript is accepted for publication, my named report including any attachments I upload will be posted on the website along with the authors' responses. I agree for my report to be made available under an Open Access Creative Commons CC-BY license (http://creativecommons.org/licenses/by/4.0/). I understand that any comments which I do not wish to be included in my named report can be included as confidential comments to the editors, which will not be published.

Choose an item.

To further support our reviewers, we have joined with Publons, where you can gain additional credit to further highlight your hard work (see: https://publons.com/journal/530/gigascience). On publication of this paper, your review will be automatically added to Publons, you can then choose whether or not to claim your Publons credit. I understand this statement.

Yes Choose an item.
