# Supplementary material for: An ecosystem for producing and sharing metadata within the web of FAIR Data
Source: Gigascience. 2025 Jan 8;14:giae111. doi: 10.1093/gigascience/giae111 (PMC11707607; doi:10.1093/gigascience/giae111)
Supplement: giae111_Reviewer_2_Report_Original_Submission — Philippe Rocca-Serra -- 6/23/2024 Reviewed [file giae111_reviewer_2_report_original_submission.pdf]

Reviewer Report

Title: An ecosystem for producing and sharing metadata within the web of FAIR Data

Version: Original Submission **Date:** 6/23/2024

Reviewer name: Philippe Rocca-Serra

Reviewer Comments to Author:

The reviewer thank the authors for their work, which presents a set of software components working together for the creation, editing and sharing of metadata. All code is made available via github code repository and is supported by extensive documentation.Main comments:A large amount of work and engineering went into the Maggot ecosystem. The communication could however be improved by clarifying the narrative.The focus of the Maggot is 'generic metadata' reporting as opposed to domain specific metadata (e.g., metadata specific to report experimental details, instrumentation specifics, experimental results).*In the 'Design Consideration' section, the authors highlight their structured approach starting with descriptor identification, terminology definition or adoption and finally standard selection.Can the authors clarify the following sentence "Rather than mandating data conformity, the schema should be flexible to accommodate pre-existing data. " in the second paragraph of that section?In the following section about 'controlled terminologies', can Maggot be set to terminology services based on Bioportal? for instance, is it possible to interact with EMBL-EBI Ontology Lookup Service or to a custom service? what would be the technical requirements ? It might be helpful to provide a pointer to Maggot documentation on that topic.In the next paragraph, as the authors argue for raising awareness in existing resources, pointing to resources such as FAIRSharing.org, Re3data or Bioregistry.org would be useful.In the following section, can the authors specify provide an example of Core Trust seal repository have targeted ? Similarly, in the case of submitting metadata via API, which schema have need chosen or can be selected (e.g., datacite, dcat)?What is " a metadata crossing approach based on the mapping defined upstream by the data manager"? can it be simplified by saying that 'mappings are available between selected models"? How many models have been mapped and are available out of the box?Providing a pointer to the extensive maggot documentation would be a nice addition: https://inrae.github.io/pgd-mmdt/chats/chat4/The last paragraph of the section seems to somehow contradict an earlier section claiming that "creating metadata should not compel data producers to engage in training in topics or concepts outside their expertise, such as FAIR principles, the semantic web, or metadata schema, unless they choose to do so" with the following sentence "Unavoidably, thedevelopment and dissemination of metadata, involving a metadata-centric culture, underline the need for ongoing training initiatives and data stewards are pivotal in educating data producers".*Result section:s/supporting the wide data diversity range within a collective./ supporting a community's varied annotation needs/The first 2 sentences of the third paragraph to section may be at ease in the design consideration section, as it describes to decoupling a storage and metadata creation.The authors introduced Maggot as tool specifically targeting 'generic metadata'. However, in the following sentence, they state " Maggot supports the input of both descriptive and administrative metadata for any type of data, including datasets, images, sequences, and more, with customizable field

definitions to suit diverse user requirements. Moreover, Maggot emphasises ease of use and adaptability." How can users know when they cross over into domain specific metadata?ODAM appears in figure 2. Can the authors provide more information about the relation with Maggot and ODAM when it comes to metadata description. Can Maggot be used to index metadata declared in ODAM or is maggot solely concerned with generic metadata about the ODAM resources (authors, owner, organization, terms of use)?In the fourth paragraph on the section, it could be beneficial to introduce the selection of OAI-PMH earlier and then explain the benefit it provides in terms of long term preservation and/or migration away from Maggot.Does Maggot support the export of metadata as schema.org based json-ld payloads natively?*Implementation and Documentation section:Can the authors expand on the issue of access to controlled vocabularies via APIs? In particular, if any caching mechanism is in place and if different versions of specific vocabularies can be fetched or specified?The authors should be commanded for the extensive mkdocs based documentations. It may be nice to amend some of the figures (e.g. figure 3, https://inrae.github.io/pgd-mmdt/publish/) so they can highlight the availability of that excellent resource for the readership*Conclusion Section:The first sentence feels a bit too long. break in 2 and push the "frictionless FAIR metadata sharing' to another sentence. the authors should be mindful of using 'frictionless' in this context due to use of frictionless data framework in ODAM, as a possible source of confusion. suggestion = s/frictionless/native/The last sentence of the first section leads to the following question: Does maggot allow granular specification of terms of uses (i.e, beyond the provision of a uri to a license document) ? If so, it would nice to add a figure or code snippet showing how this is done.Minor comments:Title: What is the "web of FAIR data"? could be the title be simplified to "Maggot : ecosystem for producing and sharing metadata to support FAIR data practice "?Abstract.background: order -> 1/reporting, 2/discovery, 3/mobilisation. then "poses challenges to data producers"s/" in alignment with FAIR principles/"in accordance to the FAIR principles"/Abstract.results: "Comprehensible" - some ambiguity here as to where it should be "comprehensive" or "explicit"? Then a question about "Collaborative environment" . Does it mean that Maggot allows concurrent editing of the same document by multiple users (as in a Google document for instance)?s/for enabling/ to enable/

Level of Interest

Please indicate how interesting you found the manuscript: Choose an item.

Quality of Written English

Please indicate the quality of language in the manuscript: Choose an item.

Declaration of Competing Interests

Please complete a declaration of competing interests, considering the following questions:

- Have you in the past five years received reimbursements, fees, funding, or salary from an organisation that may in any way gain or lose financially from the publication of this manuscript, either now or in the future?
- Do you hold any stocks or shares in an organisation that may in any way gain or lose financially from the publication of this manuscript, either now or in the future?
- Do you hold or are you currently applying for any patents relating to the content of the manuscript?
- Have you received reimbursements, fees, funding, or salary from an organization that holds or has applied for patents relating to the content of the manuscript?
- Do you have any other financial competing interests?
- Do you have any non-financial competing interests in relation to this paper?

If you can answer no to all of the above, write 'I declare that I have no competing interests' below. If your reply is yes to any, please give details below.

I declare that I have no competing interests

I agree to the open peer review policy of the journal. I understand that my name will be included on my report to the authors and, if the manuscript is accepted for publication, my named report including any attachments I upload will be posted on the website along with the authors' responses. I agree for my report to be made available under an Open Access Creative Commons CC-BY license (http://creativecommons.org/licenses/by/4.0/). I understand that any comments which I do not wish to be included in my named report can be included as confidential comments to the editors, which will not be published.

Choose an item.

To further support our reviewers, we have joined with Publons, where you can gain additional credit to further highlight your hard work (see: https://publons.com/journal/530/gigascience). On publication of this paper, your review will be automatically added to Publons, you can then choose whether or not to claim your Publons credit. I understand this statement.

Yes Choose an item.
